# Relationship between muscle mass and muscle strength, and the impact of comorbidities: a population-based, cross-sectional study of older adults in the United States

**DOI:** 10.1186/1471-2318-13-74

**Published:** 2013-07-16

**Authors:** Lei Chen, David R Nelson, Yang Zhao, Zhanglin Cui, Joseph A Johnston

**Affiliations:** 1 Lilly Research Laboratories, Eli Lilly and Company, Lilly Corporate Center, Indianapolis, IN 46285, USA

**Keywords:** Muscle mass, Muscle strength, Older adults, Correlation, Comorbidities

## Abstract

**Background:**

Loss of muscle mass and muscle strength are natural consequences of the aging process, accompanied by an increased prevalence of chronic health conditions. Research suggests that in the elderly, the presence of comorbidities may impact the muscle mass/strength relationship. The objectives of this study were to characterize the muscle mass/strength relationship in older adults in the USA and to examine the impact of a variety of comorbidities on this relationship.

**Methods:**

Data were obtained from the National Health and Nutrition Examination Survey 1999–2002 databases. Subjects aged 50 years and older were included in the present study. Muscle mass was assessed by height-adjusted appendicular skeleton muscle mass (aASM) in kg/m^2^, as measured by dual-energy x-ray absorptiometry. Muscle strength was assessed via isokinetic quadriceps strength (IQS) in newton as measured by a dynamometer. The relationship between aASM and IQS was assessed adjusting for age and gender. The effects of a variety of comorbidities on IQS and/or on the relationship between IQS and aASM were assessed using multiple regression models.

**Results:**

This study included 2,647 individuals, with a mean age of 62.6 years and 52.9% of whom were female. The mean (SE) aASM (kg/m^2^) was 7.3 (0.04), and the mean (SE) IQS (newton) was 365.0 (3.00). After adjusting for age and gender, the correlation coefficient between aASM and IQS was 0.365 (P < 0.001). Diabetes, coronary heart disease/congestive heart failure (CHD/CHF), and vision problems were significant predictors of lower muscle strength (P < 0.05) in the multiple regression models that adjusted for age, gender, and aASM, and obesity significantly modified the relationship between aASM and IQS (P < 0.05).

**Conclusions:**

Among individuals aged 50 and older in the US, muscle mass and muscle strength are positively correlated, independent of the associations of age and gender with muscle mass and strength. A variety of comorbid medical conditions serve as independent predictors of lower muscle strength (e.g., diabetes, CHD/CHF, vision problems) and/or modify the relationship between muscle mass and muscle strength (e.g., obesity).

## Background

By the year 2020, there are projected to be almost 55 million Americans, in the USA, over the age of 65 and more than 6 million over the age of 85 [1]. One consequence of the aging of the population will be an increase in the number of Americans who will experience the predictable loss of muscle mass and muscle strength that occurs with aging [2]. While not necessarily pathological, this age-related loss of muscle mass and strength has been shown to have important health consequences [3,4], predisposing the elderly to falls and contributing to other functional limitations [5,6].

Although the positive correlation between muscle mass and muscle strength has been well established [2,7-11], it is not clear whether higher muscle mass necessarily translates into greater muscle strength or whether gains in muscle strength cannot be achieved without corresponding gains in muscle mass. For example, Park et al. (2006) and colleagues found in the Health, Aging, and Body Composition (Health ABC) study that, while older adults with type II diabetes had higher muscle mass compared with their non-diabetic counterparts, they did not exhibit greater muscle strength [12]. Similarly, Raue and colleagues, studying the impact of conditioning on muscle mass and muscle strength in octogenarian women, found that whole muscle strength increased with resistance training, while muscle mass did not increase [13].

A number of clinical conditions have also been shown to correlate with muscle mass and/or strength, and it is possible that heterogeneity among older adults with respect to these conditions, many of which are increasingly prevalent with age [14], may contribute to the indeterminate relationship between muscle mass and muscle strength. While several studies corroborate the impact of comorbid conditions such as diabetes, obesity, and osteoarthritis on muscle mass, muscle strength, or related functional outcomes [12,15-18], no study has systematically explored the complex interactions between these conditions and muscle mass and strength.

In the present study, we aimed to better understand the relationship between muscle mass and muscle strength in US older adults, and to examine the impact of a variety of comorbidities on muscle strength and the relationship between muscle mass and muscle strength. Our specific objectives were as follows: (1) to describe muscle mass and muscle strength, stratified by age and gender; (2) to assess the degree to which muscle mass and strength are correlated, adjusting for the effects of age and gender; and (3) to explore the impact of a variety of comorbid conditions on muscle strength and/or the relationship between muscle mass and strength. For the latter objective, our general hypothesis was that certain factors, such as obesity and arthritis, might limit the degree to which higher muscle mass translates into greater muscle strength. Insights gained from these analyses could aid in the management of patients, who have muscle atrophy or wasting by providing norms and targets for rehabilitation and by raising awareness of the influence of other comorbid conditions.

## Methods

### Study sample

This was a cross-sectional, population-based study that used data from men and women 50 years and older who were enrolled in the National Health and Nutrition Examination Survey (NHANES) 1999–2002. The NHANES performs a continuous, nationally representative health survey of the civilian, non-institutionalized U.S. population and collect data on approximately 5,000 persons each year from in-person interviews, physical examinations, and medical tests. To produce nationally representative estimates, NHANES has adopted a complex, multistage, probability sampling design, and the survey oversamples certain ethnic minorities and individuals aged 60 years and older [19]. In 1999, NHANES began performing dual-energy x-ray absorptiometry (DXA) whole body examinations, and providing data on body composition, such as lean mass for total body and for each arm and leg, head, and trunk. The DXA equipment used was a Hologic QDR 4500A fan beam densitometer [20]. During 1999–2002, NHANES also collected data on muscle strength as measured by the isokinetic strength of the knee extensors using a Kin Com MP dynamometer [21], which enabled the study of both muscle mass and muscle strength. A total of 2,647 men and women, 50 years of age and older, with muscle mass and muscle strength data available from the NHANES 1999–2002, were included in the present study.

### Measures

Muscle mass was assessed using lean mass, excluding bone mineral content (BMC), obtained from DXA scan. In previous research, lean mass, excluding BMC, has been shown to validly represent skeletal muscle mass in the extremities [22]. In our study, appendicular skeletal muscle mass (ASM) for each subject was calculated as the sum of lean mass, excluding BMC, in both arms and both legs. A height-adjusted index (aASM) was then calculated by dividing a subject’s ASM in kilograms (kg) by the subject’s height in meters squared (m^2^) [23,24]. This index, expressed in kg/m^2^, was the muscle mass measure used for all analyses.

Muscle strength was established using measurement of the isokinetic strength of the knee extensors at peak force (isokinetic quadriceps strength [IQS]), in newtons [21]. There is evidence that lower extremity strength is associated with physical function, an important component of quality of life [25]. In NHANES 1999–2002, a total of six measurements of right quadriceps muscle strength were taken, three warm-up trial measurements followed by three outcome measurements. If a survey participant completed 4 to 6 measures, the highest peak force was selected from trials 4 to 6. If a survey participant had completed fewer than 4 trials, the highest peak force from the warm-up trials was selected. The IQS, expressed as newtons at one speed (60 degrees/second) on the right side, was the muscle strength measure used for all analyses.

Comorbidity data were obtained from self-reported personal interview questionnaire data collected in the NHANES. Presence of a variety of health conditions, which potentially affect muscle mass and/or strength, was assessed using data from the medical conditions/history questionnaire, including the following: diabetes; coronary heart disease (CHD) (including heart attack/myocardial infarction)/congestive heart failure (CHF);vision problems; obesity; arthritis (including both rheumatoid and osteoarthritis); asthma; osteoporosis; cancer (multiple varieties, excluding skin cancer); and chronic bronchitis/emphysema. Body mass index (BMI) was also investigated and defined as weight (kg) divided by height squared (m^2^). Consistent with the recommendation from Center for Disease Control and Prevention [26], the following four categories of BMI were adopted in this study: BMI < 18.5 kg/m^2^ (underweight), 18.5-24.99 (healthy weight), 25–29.99 (overweight), and ≥30 (obese).

### Statistical analyses

Statistical procedures were employed that made use of the information from the complex, multistage, probability sampling design of the NHANES. Sampling weights were used to create mean estimates that were nationally representative of the older adult population, taking advantage of the oversampling of subjects in particular age, racial, and ethnic groups. All statistical analyses were design-based, except the partial correlation coefficient calculation that used raw, un-weighted data.

Distributions of aASM and IQS, by gender and age group (in five-year intervals), were presented using box plots. The box plots present the mean, 5^th^, 25^th^, 50^th^ (median), 75^th^ and 95^th^ percentiles. The Pearson partial correlation coefficient, controlling for age and gender, was used to examine the association between aASM and IQS.

Multiple regression models, using survey strata and weighting, were conducted to examine the effect of each comorbidity on IQS and on the relationship between aASM and IQS. We first constructed a linear regression model on IQS with aASM as an exploratory variable, adjusting for age and gender (Model 1). Next, for each comorbidity, we built two additional linear regression models on IQS, one with the studied comorbidity as an exploratory variable, adjusting for aASM, age and gender (Model 2), and another with all variables in Model 2, plus the interaction between aASM and the comorbidity of interest (Model 3). The regression coefficients for aASM and the studied comorbidity were presented, including the 95% confidence intervals (CIs), along with the significance level of the interaction term from Model 3. Where results suggested a significant interaction between aASM and a given comorbidity, the relationship between aASM and IQS was graphically presented via scatter plot by using the raw non-weighted data, along with the age- and gender-adjusted regression lines, via Model 3 and stratifying on comorbidity status. In addition, subgroup analyses of linear regression models (Model 1) were fit within comorbidity subgroups (e.g., arthritis and non-arthritis subgroups), adjusting for age and gender.

All analyses were conducted using SAS®, version 9.2 survey procedures (SAS Institute, Cary, North Carolina, USA). All hypothesis tests were 2-tailed with a significance level of 0.05, and regression coefficients were deemed significantly different if the corresponding 95% CIs were non-overlapping.

## Results

Population characteristics are presented in Table 1. The sample represented a population with a mean (SE) age of 62.6(0.32) years of age, 52.9% female and 80.9% non-Hispanic white. The mean (SE) aASM was 7.3 (0.04) kg/m^2^, and the mean (SE) IQS was 365.0 (3.0) newtons.

**Table 1:** Baseline study sample characteristics

**Characteristics**	**Mean (SE) or n(%) of Population **^**a**^
Age (years), mean (SE)	62.6 (0.32 )
Age (years), by category	
50-54	497 (27.4%)
55-59	326 (18.4%)
60-64	518 (15.7%)
65-69	409 (13.3%)
70-74	369 (10.9%)
75-79	230 (7.6%)
80+	298 (6.7%)
Gender	
Male	1334 (47.1%)
Female	1313 (52.9%)
Race/Ethnicity	
Non-Hispanic White	1528 (80.9%)
Mexican American	517 (3.3%)
Other Hispanic	120 (5.1%)
Non-Hispanic Black	413 (7.4%)
Other Race - Including Multiracial	69 (3.2%)
Body Mass Index (kg/m^2^)	
<18.5	34 (1.2%)
18.5-24.99	739 (29.2%)
25-29.99	1103 (40.5%)
≥30	769 (29.0%)
aASM (kg/m^2^), mean (SE)	7.3 (0.04)
IQS (newton), mean (SE)	365.0 (3.00)

**Abbreviations:***aASM* height-adjusted appendicular skeleton muscle mass, *IQS* isokinetic quadriceps strength.

^a^All percentage and mean estimates were weighted according to sampling design.

**Figure 1:**
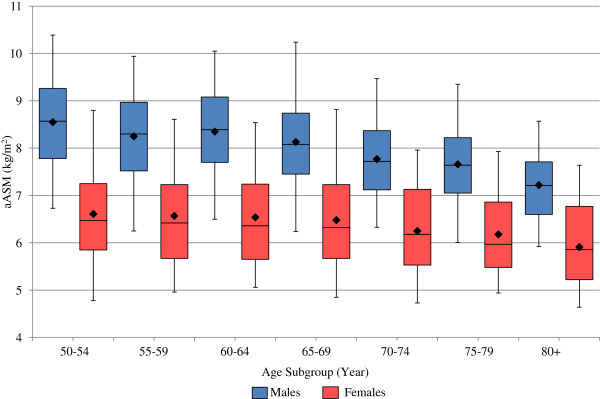
**Distribution of muscle mass by age and gender.** Lower half of boxplot depicts 25th percentile, upper half of boxplot represents 75th percentile, horizontal line dividing upper and lower half of boxplot represents median, upper whisker represents 95th percentile, lower whisker represents 5th percentile; and solid diamond in boxplot represents mean. aASM = height-adjusted appendicular skeleton muscle mass.

**Figure 2:**
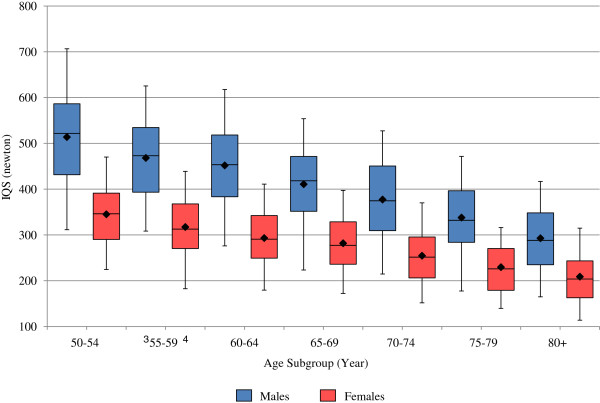
**Distribution of muscle strength by age and gender.** Lower half of boxplot depicts 25th percentile, upper half of boxplot represents 75th percentile, horizontal line dividing upper and lower half of boxplot represents median, upper whisker represents 95th percentile, lower whisker represents 5th percentile; and solid diamond in boxplot represents mean. IQS = Isokinetic quadriceps strength.

The distributions of aASM and IQS, stratified by gender and age, are presented in Figure 1 and Figure 2, respectively. Both aASM and IQS tend to decrease with age in older adult men and women, and women appear to have a lower mean aASM and IQS than men within the same age group. In men, aASM appears to decrease steadily after age 65, while a similar decrease in women occurs somewhat later (Figure 1). Mean IQS tends to decline in both men and women as early as the starting age of the study cohort (the sixth decade of life), with the IQS decline in men appearing somewhat steeper than for women.

The correlation coefficient between aASM and IQS, after adjusting for age and gender, was 0.365 (P < 0.001); in other words, the coefficient of partial determination was 0.133 (0.365^2^) [27], which indicates that aASM explained 13.3% of variance in IQS, independent of the effect of age and gender. Results from the multiple linear regression models, presented in Table 2, provided additional statistically significant evidence that supports the association between aASM and IQS. After adjusting for age and gender, the regression coefficient for aASM was 31.6 (95% CI: 27.2,36.0), representing, on average, a 31.6 (95% CI: 27.2,-36.0) newton increase in IQS for every 1 unit (kg/m^2^) increase in aASM. Age, gender, and aASM, together, explained 59.9% of the variance in IQS.

**Table 2:** **Results of multiple linear regression modeling on muscle strength**^**a**^

**Exploratory variable**	**Number of patients with comorbidity (%)**^**b**^	**Regression coefficient for aASM (95% CI)**^**d**^	**Regression coefficient for exploratory variable(95% CI)**^**d**^	**Significant level of the interaction effect (aASM by studied variable)**^**e**^
aASM^c^	—	31.6 (27.2, 36.0)***	—	—
Diabetes	364 (10%)	32.7(28.3, 37.0)***	−27.1 (12.0, 42.1)**	NS
Coronary Heart Disease/Congestive Heart Failure	177 (10%)	34.5(29.3, 39.8)***	−20.7 (−36.1, -5.3)*	NS
Vision Problem	366 (19%)	34.0(28.7, 39.2)***	−20.1 (−34.4, -5.7)*	NS
BMI	—	46.4 (38.1, 54.7)***	−3.7 (−5.4, -2.1)**	*
Arthritis	638 (39%)	35.1(29.9, 40.4)***	−19.6 (−27.4, -11.8)***	*
Asthma	158 (11%)	34.1 (28.8, 39.5)***	−6.4 (−26.3, 13.6)	**
Osteoporosis	189 (8%)	31.8(27.4, 36.2)***	−12.3 (−22.0, -2.6)*	**
Cancer	222 (14%)	34.3(28.9, 39.8)***	6.7 (−6.1, 19.6)	NS
Chronic Bronchitis / Emphysema	136 (9%)	34.0(28.7, 39.4)***	−9.2 (−20.6, 2.3)	NS

**Abbreviations:***aASM* height-adjusted appendicular skeleton muscle mass, *BMD* bone mass density, *BMI* body mass index, *CI* confidence interval, *NS* not significant (P > 0.05).

^a^All estimates weighted according to sampling design.

^b^Proportions are calculated based on subjects with non-missing data.

^c^Model 1: Exploratory variables include aASM, age, and gender.

^d^Model 2: Exploratory variables include the studied variable, aASM, age, and gender.

^e^Model 3: Exploratory variables include the studied variable, aASM, studied variable by aASM interaction, age, and gender.

*P < 0.05, **P < 0.01, ***P < 0.001.

Results from the multiple regression models that incorporated each of the comorbidities suggested that diabetes, CHD/CHF, and vision problems had significant negative main effects on IQS (P < 0.05), while corresponding interaction effects between the comorbidity and aASM were not statistically significant (P > 0.05) (Table 2). Specifically, individuals with diabetes, CHD/CHF, and vision problems demonstrated an IQS, on average, of −27.1 (95% CI: -42.1,-12.0), -20.7 (95% CI: -36.1,-5.3) and −20.1 (95% CI: -34.4, -5.7) newtons less, respectively, than the age, gender, and aASM comparable older adults without these comorbid conditions.

Body mass index, arthritis, and osteoporosis had both significant main effects and interaction effects on IQS, while asthma had a significant interaction effect, but an insignificant main effect on IQS, which made the corresponding regression coefficients less directly interpretable. Cancer and bronchitis/emphysema had neither significant main nor interaction effects.

**Figure 3:**
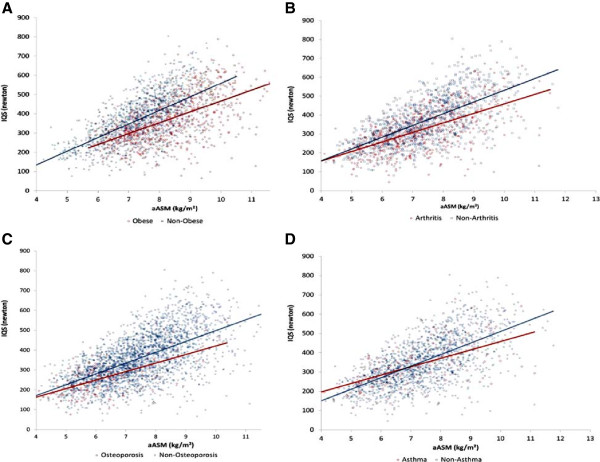
**Correlation between muscle mass and muscle strength: the modification effect of comorbidities (A. Obesity, B. Arthritis, C. Asthma, D. Osteoporosis).** aASM = height-adjusted appendicular skeleton muscle mass; IQS = isokinetic quadriceps strength.

The relationship between muscle mass and IQS among subgroups with and without the comorbidities for which there were significant interaction effects (e.g., obesity, arthritis, asthma, and osteoporosis) is shown visually in Figure 3. Subgroup analyses suggested that the regression slopes for aASM were comparable among underweight (47.9 [95% CI: 20.4,74.0], healthy weight (54.4 [95% CI: 42.3,66.5]) and overweight subgroups (47.4 [95% CI: 38.5,56.4]), while the slope for the obese subgroup (23.8 [95% CI: 13.1,34.4]) differed significantly from that of both the healthy weight and overweight subgroups. As such, for simplicity, the influence of weight was shown in the Figure 3A by stratifying subjects into only two subgroups, obese and non-obese. As shown in this figure, obese subjects (red diamonds) tend to have, on average, lower strength at a given aASM value compared to non-obese subjects (blue squares), and the regression line for this subgroup (red line) is consistently below that for the non-obese subgroup.

In Figure 3B, the regression line for the non-arthritis subgroup was generally higher than that for the arthritis subgroup. While the difference in the slopes of these regression lines was not statistically significant, the regression lines can be seen to diverge as aASM increases and to become closer and eventually to cross at lower values of aASM. A similar pattern was seen for subgroups with and without osteoporosis and asthma (Figure 3C and 3D).

## Discussion

In this cross-sectional, population-based study of older US adults, we described the distribution of muscle mass and muscle strength stratified by age and gender. We found that muscle mass and muscle strength were positively correlated after adjusting for age and gender. We also found that certain comorbidities had significant impacts on muscle strength and/or on the relationship between muscle mass and muscle strength.

To our knowledge, this is the first study to characterize the distribution of height-adjusted appendicular skeletal muscle mass in a nationally representative sample of older adults in the US. Kelly et al. (2009) provided nationally representative reference data on DXA body composition reference values, but did not include reference data on lean mass exclusive of bone mineral content, which is considered a better representation of skeletal muscle mass [24]. The distributions of aASM and IQS can serve as reference values for US older adults and may prove useful in the evaluation of abnormalities involving muscle mass and muscle strength.

In this study, we observed that both muscle mass and muscle strength declined with advancing age in older adults, and that women have a relatively lower muscle mass and strength compared with men of the same age, and these findings are consistent with previous research [8-10,28-30]. Our observation that age-associated decrements were more pronounced for muscle strength than for muscle mass corroborates the finding from a 3-year longitudinal study of 1,880 older adults; this finding showed that muscle quality (defined as muscle strength per unit of muscle mass) declines with advancing age [31].

This study quantified the positive association between muscle mass and muscle strength in older adults, and found that muscle mass explained about 13% of variance in muscle strength, independent of age and gender. After adjusting for age and gender, every one unit increase in aASM was associated with a corresponding 31.6 newton increase in IQS. As these results are derived from a cross-sectional study, any causal inference is precluded. Nevertheless, these data underscore the need for further prospective research that evaluates the downstream effects of interventions, which are aimed at preserving muscle mass, on muscle strength and physical function.

Our study also revealed that certain comorbidities impact muscle strength or the muscle mass/strength relationship. Diabetes, CHD/CHF, and vision problems were independent predictors of low muscle strength, which suggests that individuals with these comorbidities have, on average, lower muscle strength compared with individuals of similar age, gender, and muscle mass. Our finding regarding the effect of diabetes is consistent with work conducted by Park et al. (2006), wherein diabetics in the Health ABC Study were observed to have poorer muscle quality than non-diabetics [12]. Older diabetic men and women in that study exhibited greater muscle mass without a corresponding increase in muscle strength relative to non-diabetic older adults. Research in exercise physiology has established a robust dose–response relationship between physical exercise and muscular adaptation, which leads to increased motor unit activation and a proportionately greater increase in muscle strength than in muscle mass [32]. Conversely, decreased physical activity is a known risk factor for diabetes [33], and diabetics, on average, are likely to exhibit poorer muscle quality relative to their non-diabetic peers. In addition, vascular and metabolic deficits (e.g., decreased oxygen supply or impaired glucose extraction and utilization by muscle) may also contribute to physiologic impairment of muscle cells in diabetics, which results in diminished muscle strength. Deconditioning, likely, also contributes to the decrease in muscle strength that is observed in subjects with CHD/CHF, vision problems, and obesity, as these groups may be more limited in their ability to engage in regular physical activity. In addition, the increase in intramyocellular fat, which is seen in obese subjects, may also adversely affect muscle quality and force production, although the mechanism for this result is unclear [12].

While arthritis, asthma and osteoporosis all appeared to alter the relationship between muscle mass and muscle strength to some extent, interpretation of these effects is challenging due to the significant interaction effects observed. It is possible that these conditions are meaningful effect modifiers, but the results from our subgroup analyses (e.g., absence of statistically significantly differences in regression line slopes) failed to establish this. It may be that these effects are relatively marginal or perhaps more complex (e.g., non-linear), thus, requiring a larger sample size or more complex modeling approaches. Further research is warranted to better understand the effects of these comorbidities.

These findings should be interpreted in the context of study limitations. First, the cross-sectional nature of the data limits our ability to do more than describe asso-ciations, and future longitudinal research is needed to better understand the causal nature of these associations. Second, comorbidities were based upon self-report, rather than on formal clinical assessment, which raises concerns about recall and other biases. Finally, comorbidities were examined individually; whereas, in many cases, multiple comorbidities may co-occur. Unfortunately, incorporating additional two-way or three-way interaction effects would have significantly impacted the power available to draw meaningful conclusions.

## Conclusions

In conclusion, among individuals aged 50 and older in the US, muscle mass and muscle strength are positively correlated, independently of the associations of age and gender with muscle mass and strength. Certain comorbid medical conditions serve as independent predictors of lower muscle strength (e.g., diabetes, CHD/CHF, vision problems) or modify the relationship between muscle mass and muscle strength (e.g., obesity).

## Competing interests

All authors are full-time employees and stockholders of Eli Lilly and Company.

## Authors’ contributions

LC research project conception, organization, and execution; statistical analysis design; and manuscript drafting, review, and critique. DN data acquisition, statistical analysis design and execution, and manuscript review and critique. YZ research project conception and organization, and manuscript review and critique. ZC manuscript review and critique. JJ research project conception; statistical analysis design; and manuscript drafting, review, and critique. All authors read and approved the final manuscript.

## Pre-publication history

The pre-publication history for this paper can be accessed here:

http://www.biomedcentral.com/1471-2318/13/74/prepub
